# Kitasato symposium 2011: translational prospects for cytokines

**DOI:** 10.1186/ar3403

**Published:** 2011-09-16

**Authors:** Gerd R Burmester, Peter E Lipsky, Thomas Dörner

**Affiliations:** 1 Dept. Medicine/Rheumatology and Clinical Immunology Charite University Medicine, Berlin, Germany; 2 Charlottesville, NC, USA

## 

After successful meetings in 2009 and 2010, an international faculty of largely immunologists and rheumatologists will gather in Potsdam on September 22^nd ^and 23^rd^, 2011 to discuss the impact of cytokines in health and their contributions to autoimmunity in a symposium named after Shibasaburo Kitasato (1853 - 1931), who worked in Berlin between 1885 and 1892. During this rather short time, he together with Emil von Behring discovered the causative pathogens of tetanus and diphtheria and contributed substantially to our basic understanding of the interaction of the immune system and invading pathogenic microorganisms In keeping with the tradition of Kitasato, a major theme of the symposium will be the translation of basic science principles into understanding human disease. The keynote lecture of the 2011 Kitasato symposium will be delivered by Antonio Lanzevecchia (Belinzona/Switzerland) who has contributed many novel insights into understanding of immune regulation and host defense. His lecture is entitled "Dissecting the human immune response to pathogens".

This year's Kitasato Symposium will be a joint meeting with the Research Collaborative Consortium (Sonderforschungsbereich) 650 "Cellular approaches to the suppression of unwanted immune reactions - from bench to bedside". As in previous years, the Kitasato symposium will focus on mechanisms of autoimmunity and tolerance emphasizing the role of cytokines. A deeper understanding of these aspects and adapyive and innate immunity has paved the way to innovative therapies for autoimmune disease within the last decade, especially in rheumatoid arthritis (inhibition of TNF, IL-1 and IL-6R) and very recently in systemic lupus erythematosus (BAFF/BLyS blockade).

In specific sessions, the role of tolerance in autoimmunity as well as transplantation, signaling pathways in cytokine stimulation, the analysis of new cytokine targets, and the translational of cytokine biology into human disease will be discussed. The Symposium will especially focus on novel developments within the last few years with the promise of yielding new targets for therapy. In addition, insights on disease biology developing from the clinical use of biologis will be highlighted.

It is the promise of the meeting to provide new perspectives of basic, translational and clinical research in the field serving the ultimate goal of improving the treatment of patients. The collection of the individual contributions is summarized in the following abstract supplement.

